# Perennial Kernza cropping promotes rhizosphere microbiome stability and endophyte recruitment compared to annual wheat

**DOI:** 10.1186/s40793-025-00794-3

**Published:** 2025-11-07

**Authors:** Sulemana Issifu, Arval Viji Elango, Kristina Michl, Christophe David, Tomislav Cernava, Roland C. Wilhelm, Frank Rasche

**Affiliations:** 1 Institute of Agricultural Sciences in the Tropics (Hans-Ruthenberg-Institute), University of Hohenheim, Garbenstr. 13, 70599 Stuttgart, Germany; 2 Department of Agronomy, Lilly Hall of Life Sciences, Purdue University, 915 Mitch Daniels Blvd, Indiana 47907 West Lafayette, USA; 3 Institute of Environmental Biotechnology, Graz University of Technology, 8010 Graz, Austria; 4 Department of Agroecosystems, Environment and Production, ISARA, Lyon Cedex 07, France; 5 School of Biological Sciences, Faculty of Environmental and Life Sciences, University of Southampton, Southampton, SO171BJ UK; 6 Present Address: International Institute of Tropical Agriculture (IITA), P.O. Box 30772-00100, Nairobi, Kenya

**Keywords:** Wheat, Bacterial community, Agricultural microbiology, Endophytes, Kernza, Perennial plants, Rhizosphere

## Abstract

**Background:**

Perennial cropping systems are increasingly recognized for their potential to enhance microbial biodiversity and beneficial soil functions compared to annual crops. The impact of perennialization on the rhizomicrobiome and endophyte community was assessed by comparing intermediate wheatgrass (*Thinopyrum intermedium*, commercialized as Kernza®, hereafter called ‘Kernza‘) and annual wheat (*Triticum aestivum*) associated communities across a north–south European agroclimatic gradient (Sweden, Belgium, and France) over two growing seasons and at two depths.

**Results:**

Between the 2 years, the Kernza-associated rhizomicrobiome was more stable and exhibited greater homogeneity across depths compared to annual wheat. Kernza harboured a significantly more diverse set of crop-associated amplicon sequence variants (ASVs) and had a higher number of core ASVs than annual wheat. Furthermore, Kernza had a significantly higher proportion of rhizobacterial populations in root tissues than annual wheat. Environment-wide association analyses revealed that the Kernza rhizosphere had higher proportions of grassland-associated and rhizosphere-dwelling microbiomes compared to annual wheat. Despite these noteworthy differences, the greatest variation in the rhizomicrobiome composition was driven by factors such as country, year, and depth, rather than crop type. For instance, Actinobacteriota dominated rhizobacterial communities in both Kernza and annual wheat.

**Conclusions:**

Overall, Kernza conferred modest yet clear improvements in rhizomicrobiome community stability and selective endophyte recruitment, supporting its ability to enhance sustainable, microbially-mediated soil functions. Moreover, Kernza hosted significant grassland-associated taxa, suggesting a similarity between Kernza fields and grassland ecosystems.

**Supplementary Information:**

The online version contains supplementary material available at 10.1186/s40793-025-00794-3.

## Background

Lands cultivated for agriculture (‘agroecosystems’) cover about 38% of Earth’s land surface [1]. Population growth is heightening the pressure on sustainable land management, as agricultural intensification and expansion significantly contribute to climate change [2], biodiversity loss [3, 4], and declining soil fertility [5], amongst other challenges. Cropping systems vary in their ability to sustain or enhance natural processes (nature-based solutions) that help mitigate these harmful effects [6–9]. Cropping systems that leverage relevant biochemical activities of soil-borne microorganisms, particularly in the rhizosphere, can be used to foster biodiversity and supplement plant nutrition or other agroecosystem services [10, 11]. Thus, the management of beneficial interactions between crop, cropping system, and rhizosphere microbial communities (‘rhizomicrobiome’) is a target for advancing efficient, sustainable agricultural land use.

The choice of cropping system is among the most influential decisions on the ecological impacts of agricultural management [12]. Cropping systems dictate the degree of disturbance, plant cover, and a range of environmental conditions that influence rhizomicrobiome structure and function [11, 13]. For example, increasing the number of crops in rotation in maize production can alter rhizomicrobiome composition and recruitment of root endophytes [12]. Rhizomicrobiome diversity can also vary by genotype, for example in maize [14], resulting from crop- and cropping system-specific changes in root exudation, root architecture, and litter quality [15, 16]. While crops and cropping systems exert extensive influence on rhizomicrobiome, the rhizomicrobiome, in turn, influences soil nutrient cycling, soil formation, and plant health [14, 17, 18]. These observations inform our expectation that cropping systems which utilize perennial plants should exert a persistent, and strong influence on rhizomicrobiome biodiversity, function, and the complexity of the soil trophic web.

Incorporating perennial crops into cropping systems, a practice known as 'perennialization', offers a way to enhance agroecological functions and biodiversity [19, 20]. A meta-analysis of tropical agroecosystems revealed that perennial crops mitigated the loss of—and in some cases enhanced—microbial biodiversity, while annual crops drastically reduced biodiversity [21]. Perennial crops develop extensive root systems which can exert a larger and sustained influence over rhizomicrobiome activity. Root systems of perennial crops shape rhizomicrobiome composition, biomass, diversity, and activity, in large part due to changes in the quantity and quality of carbon inputs which may be higher than in annual crops [22–24]. Perennialization produces broad shifts in soil metabolite pools in fields between annual (*Triticum durum*) and perennial (*Thinopyrum intermedium)* wheat cropping systems, producing corresponding shifts in the soil microbiome from which the rhizomicrobiome and root microbiome assemble [22, 25]. These influences may shape the performance of perennial crops in ways that enhance or decrease (e.g., elevate pathogen burden) crop performance over time.

*Thinopyrum intermedium*, an intermediate wheatgrass, commercialized as Kernza® (hereafter called ‘Kernza’), is a perennial cereal grain crop that provides multiple ecosystem services that support sustainable grain production [26–28]. Perennial cereal grain crop species are designated as *k*-strategists due to their long life-span and efficient resource-use, a life-history strategy associated with a comparatively large investment in below-ground activities [29]. Kernza has deep roots that enhance the translocation of plant-derived carbon belowground, and sequestration of nutrients and water retention [30–32], restricting the loss of nitrogen via leaching, surface runoffs, and nitrous oxide emissions [33–35]. Crops that are *k-*strategists reportedly stabilize soil microbiome composition, leading to greater resistance to soil disturbance [36]. Microbiome stability refers to the maintenance and consistency in the composition and activity of the microbiome across spatio-temporal scales [37]. Compared to annual wheat, Kernza has been found to promote soil health by increasing populations of earthworms [38], nematodes [39, 40], and Orbatid mites [41], as well as promoting microbial activity [26]. Relative to annual wheat, studies of Kernza report higher rates of carbon accumulation [41, 42], retention of soil organic matter [26], enhanced nitrogen cycling [25, 43], and reduced soil erosion [44]. Kernza may very well yield a more stable and active rhizomicrobiome, according to its life-history traits, which may enable new sustainable management practices and serve as a breeding target during on-going domestication efforts.

Relative to annual wheat cropping systems, perennial Kernza systems have shown modest impacts on the composition and functionality of the soil and rhizosphere microbiome. Studies investigating these effects have yielded varying results, likely depending on the age of perennial plantations, soil type, and which aspect of the rhizomicrobiome were characterized. A growing consensus of research indicates that Kernza systems increase total bacterial abundance, according to quantification of the 16S rRNA gene [26, 29, 43]. The composition of bacterial endophyte communities in Kernza tissues were only significantly different from annual wheat in the root compartment, not leaves or stems, where Kernza root endophytes also exhibited greater species richness [45]. Fungal populations also exhibited higher species richness and biomass, including arbuscular mycorrhizal fungi marker lipids, in the rhizosphere of Kernza compared to annual wheat based on both internal transcribed spacer amplicon and phospholipid fatty acid analyses [29, 42]. However, rhizosphere bacterial (‘rhizobacterial’) community composition does not always vary between Kernza and annual wheat systems [42]. With field trials spanning differences in soils and climate, we sought additional evidence to weigh the influence of Kernza on the stability and composition of rhizobacterial populations, and any knock-on effects on endophyte populations.

Using the first Kernza field trials in Europe, we set out to identify the influence of perennialization on the structure and stability of rhizobacterial populations and bacterial root endophytes in comparison to annual wheat. According to prior evidence, we hypothesized that continuous Kernza cropping (1) will facilitate a higher alpha-diversity relative to annual wheat, given reports of higher root endophyte richness [45], (2) have a higher relative abundance of crop-associated rhizobacterial populations in deeper soils (25–35 cm) due to its deep rooting system, (3) have lower variation in community composition (beta-diversity) from year-to-year, and (4) recruit a higher proportion of endophyte populations from rhizobacterial populations than annual wheat due to a more enduring recruitment by roots. To test our hypotheses, we undertook an amplicon-sequencing based field survey of rhizosphere and root endophyte populations in field trials of Kernza and annual wheat (4–6 years) at three sites across an agro-climate transect (Sweden, Belgium, and France), at two depths (5–15 cm and 25–35 cm), over two growing seasons. We evaluated trends in alpha- and beta-diversity and the selection of ‘core’ populations over time and across depth in the rhizosphere and root endophytes of both crops. Additionally, we performed an environment-wide association analysis of the microbiome of each crop using the AgroEcoDB [46] to assess the generalizability of trends in rhizobacterial populations related to land use (grassland ecosystem), disturbance, rhizosphere status, and soil fertility/health. Collectively, our study sought to provide evidence of the scope and scale of influence of perennial root systems on the overall rhizomicrobiome composition and structure, stability of rhizobacterial community and its influence on the recruitment to the root endosphere.

## Methods

### Study design and conditions of study sites

The study was conducted across a European transect at field sites in Sweden (Lönnstorp; 55° 40′ 0″ N, 13° 5′ 0″ E), France (Saint Marcel Bel Accueil; 45° 34′ 5″ N, 5° 15′ 58″ E and 45° 63′ 29″ N, 5° 25′ 72″ E), and Belgium (Gembloux; 50° 34′ 0″ N, 4° 41′ 0″ E). Kernza field trials had been established in 2016 for Sweden, and in 2017 for Belgium and France. All locations were sampled in 2021 and 2022 at between 4 and 6 years old. Field sizes varied with the largest fields in Sweden (50 × 24 m; ~ 1200 m^2^), then France (40 × 18 m; ~ 720 m^2^ ha), and Belgium (7 × 2 m; ~ 14 m^2^). Kernza and annual wheat were established as monocrops. For Belgium and Sweden, four subplots had been experimentally established in randomized block designs, whereas farmers’ fields were used in France, which were pseudo replicated during sampling to capture field heterogeneity. Thus, France was considered as one plot, without experimental replicates, while each subplot was considered as a replicate in Belgium and Sweden (n = 4). Fields in each country varied in terms of agroclimatic zone, edaphic soil properties, and sampling timepoints, which occurred at different developmental stages (*ex.* tillering and flowering). Consequently, crop type, country, depth, and year were considered as distinct factors for comparisons. The differences in agro-climatic properties of each site are shown in Table 1. Additional details of field sizes, replicates, and soil and agroclimatic conditions of all study sites are reported in previous publications by Förster et al. [38] and Michl et al. [45]. Our field sites have already been investigated for the influence of perennialization on endophytic bacteria [45], fungi, and other soil macrofauna [38]. Kernza was domesticated by The Land Institute (Kansas, USA) and is currently licensed for commercial production in the USA and Canada.

**Table 1:** Description of study sites and soil edaphic properties

Location	Climate	Soil type	Crop	Year of establishment	Soil Coverage (%)	Depth (cm)	Soil texture	pH	TOC (g/kg)	TN (g/kg)	C:N ratio	WHC (Vol-%)
France (Saint Marcel, Bel Accueil)	Temperate climateRainfall: 842.60 mm Temperature: 11.64 °C	Gleyic Eutric Fluvisol (Loamic, Humic)	Wheat	–	2.61	5–15	Clayey loam	7.5	52.16	4.28	19.25	66
						25–35	Clayey loam	7.5	46.96	3.82	19.57	74
		Gleyic Eutric Fluvisol (Loamic, Humic)	Kernza	2018	76.6	5–15	Clayey loam	7.4	31.76	2.93	12.29	74
						25–35	Clayey loam	7.5	25.54	2.13	16.05	66
Belgium (Gembloux)	Temperate maritime climate Rainfall: 898.90 mm Temperature: 10.67 °C	Haplic Luvisol (Loamic, Humic)	Wheat	–	20.73	5–15	Silty loam	6.9	9.97	1.14	9.26	64
						25–35	Silty loam	7	7.18	0.89	8.58	63
		Haplic Luvisol (Loamic, Humic)	Kernza	2019	82.71	5–15	Silty loam	6.8	13.64	1.26	10.98	65
						25–35	Silty loam	6.8	10.25	1.01	10.14	65
Sweden (Lönnstorp)	Temperate climate Rainfall: 653.66 mm Temperature: 3.18 °C	Eutric Stagnic Cambisol (Loamic, Humic)	Wheat	–	68.66	5–15	Clayey loam	5.8	16.41	1.84	9.06	66
						25–35	Clayey loam	6.2	7.78	1.32	5.88	66
		Eutric Stagnic Cambisol (Loamic, Humic)	Kernza	2016	84.84	5–15	Clayey loam	5.7	16.42	1.72	9.55	66
						25–35	Clayey loam	6.4	10.19	1	8.54	66

### Rhizosphere soil sampling and processing

Rhizosphere soil samples were collected in June 2021 (summer) and April 2022 (spring) to capture temporal dynamics. Sampling plot size was scaled to the field size of each study site as published in our previous studies [38, 45]. At the time of sampling in 2021 and 2022, the crops were at the flowering (BBCH 61–65) and tillering (BBCH 21–29) stages, respectively. Sampling of rhizosphere soil was done by taking cores with a split tube sampler (Royal Eijkelkamp, Giesbeek, Netherlands; length: 45 cm; diameter: 5.3 cm). Within each sub/pseudo plot, five cores were taken randomly from the root zone of the crops. The cores were divided into two segments of 5–15 cm and 25–35 cm to capture the spatial dynamics within the rhizosphere. In 2021, sampling at 25–35 cm could not be taken for Sweden due to the extremely compact nature of the soil. Roots obtained from the cores were separated into their respective layers, packaged in zip-lock bags, labelled appropriately, and shipped on ice to the University of Hohenheim (Germany). Upon arrival, samples were immediately stored at -25 °C until further processing. Subsequently, soil adhering to the roots, deemed rhizosphere soil, was brushed off into new appropriately labelled zip-lock bags, and stored at − 25 °C for DNA extraction. Roots were provided to a working group focused on differences in the endophyte communities of Kernza and annual wheat, whose paired analysis has been recently published [45].

### DNA extraction, 16S rRNA amplicon sequencing, and sourcing endophyte data

DNA was extracted from soil using the FastDNA Spin Kit for Soil (MP Biomedicals GmbH, Eschwege, Germany) and DNeasy PowerSoil Pro Kit (Qiagen GmbH, Hilden, Germany) for 2021 and 2022 soil samples, respectively, according to the manufacturers’ instructions. The use of different DNA extraction kits resulted from sourcing issues during COVID-19. Any biases due to extraction were confined within each year, making comparisons among crops valid, while potentially obscuring weaker trends in populations across years. DNA extracted from each of the five cores were pooled into one DNA extract per plot (n = 4) per layer per crop (annual wheat and Kernza) for Belgium and Sweden. However, since we considered France fields as one plot for each crop (20 cores), DNA extracts were pooled for each layer making a total of two samples per crop. DNA pooling was done by first diluting DNA samples to a concentration of 10 ng µL^−1^ with PCR-grade water, transferring 5 µL from each sample into appropriately labelled 2 mL Eppendorf tube, and homogenizing by vortexing. DNA was quantified with a Nanodrop 2000 Spectrophotometer (Thermo Fisher Scientific, USA) before and after pooling. DNA samples were stored at − 25 °C until further analysis. After pooling all samples appropriately, a total of 64 total rhizosphere samples were obtained for analysis.

Amplicon sequencing was performed according to the Earth Microbiome Project protocol with a few modifications [47]. Briefly, the variable V4 region of the 16S rRNA gene was PCR amplified using the primers: 515F (5′-GTGYCAGCMGCCGCGGTAA-3′) and 806R (5′-GGACTACHVGGGTWTCTAAT-3’) [48, 49]. The primers contained 10 bp additional Golay barcodes [50] (Table S1). Reaction volume of the PCR was 25 µL containing 18.8 µL PCR-grade water, 4 µL 5 × HOT FIREPol Blend Master Mix (Solis Biodyne, Tartu, Estonia), 0.6 µL primers (10 µM), and 1 µL DNA. PCR conditions were set as follows: 15 min at 95 °C, followed by 30 cycles of 95 °C for 30 s, 60 °C for 30 s, 72 °C, and a final elongation cycle of 72 °C for 5 min. PCR reactions were performed in three replicates, with three negative controls included. Amplicons (5 µL) were verified by electrophoresis on a 1% agarose gel, using the negative controls as benchmarks to confirm the absence of contamination. The resulting products were pooled and purified using the Wizard SV Gel and PCR Clean-Up System (Promega, Madison, USA). Subsequently, amplicon concentrations were measured using the Qubit dsDNA BR Assay Kit (Thermo Fisher Scientific, Waltham, MA, USA). All samples were pooled (multiplexed) in equimolar concentrations and outsourced to Novogene (UK) for sequencing (Illumina MiSeq; 2 × 250 bp). As no contamination was observed, the negative controls were excluded from sequencing—with sequencing controls being handled by the provider.

Paired endophyte 16S rRNA gene data was sourced from the European Nucleotide Archive (accession: PRJEB74910) [45]) which was derived from root samples collected from the same samples used in the current study. Michl et al. [45] found that roots were the only compartment where endophyte populations differed between Kernza and annual wheat. A targeted re-analysis of this endophyte dataset was used to evaluate the degree of overlap (or ‘recruitment’) of endophyte populations from rhizobacterial populations. Furthermore, we delved deeper into which specific populations differed between the two crops, an analysis which was not presented by Michl et al. [45]. The root endophyte data was generated from both years at only 5–15 cm depths from 20 plants per field per crop type, yielding a total of 240 paired samples. Each endophyte sample was sequenced unlike our rhizosphere samples which were pooled. Consequently, each rhizosphere sample corresponds to multiple root endosphere samples which were taken from the same spots (Table S1).

### Bioinformatics and statistics

Samples were demultiplexed and processed using the QIIME2 pipeline v. 2023.9 [51] using cutadapt to remove barcodes and primer sequences [52]. DADA2 (v. 1.10) was used for quality filtering of the data, denoising, removing chimeras, assigning amplicon sequence variants (ASVs) based on the exact sequence variants [53]. The first five nucleotides of all amplicon sequences were trimmed to enable direct comparison to studies in the AgroEcoDB [46], used to identify environment-wide associations of Kernza- or annual wheat-associated ASVs (details below). Taxonomic classification was performed with the ‘q2-feature-classifier’ utilizing the Greengene2 database [54]. ASVs were filtered for sparsity (< 3 samples were removed) and low abundant taxa (< 1% of total reads) and if ASVs were unclassified or classified as chloroplasts or mitochondria. After quality filtering, a total of 5,406,896 sequences (87.1%) and 20,074 ASVs (76.1%) remained. Sequencing depth per sample ranged from 1,060 to 135,754 reads, with a mean of 73,583 ± 47,210 reads.

Community analyses and statistics were performed with R (v. 4.3.3) in R studio (v. 2023.06.1) with dependency on the package *phyloseq* [55]. The proportional abundance of ASVs was calculated as the read counts per thousand reads and, in places, also presented as the aggregate percent abundance at various taxonomic ranks—including Phylum, Order, or Genus. To identify ASVs associated with study factors (*eg.* crop type), we performed indicator species analysis (n_perm_ = 10,000) using *indicspecies* (v. 1.7.14) [56] using the merged data for all countries, years, and depths. Indicator ASVs which were non-significant (*P* < 0.05) and with lower indicator values (< 0.35) were removed from the analysis. Alpha-diversity was measured as observed species richness, Chao1 index, Simpson diversity index, and Shannon diversity using normalized data (counts per thousand). Beta-diversity was evaluated using Bray–Curtis dissimilarity which was subjected to PERMANOVA (n_perm_ = 999) to test for the significance of study factors using *adonis2* from the R package *vegan* (v. 2.6.1) [57]. Trends in beta-diversity were visualized on a two-dimensional plane using non-metric multidimensional scaling (NMDS) based on the Bray–Curtis dissimilarity among communities.

We performed a range of focused analyses of trends in the rhizomicrobiome and endophyte microbiome to test our hypotheses. We contrasted the dissimilarity in rhizomicrobiome composition from year-to-year, between depths, and across study sites by comparing the mean pairwise Bray–Curtis dissimilarity (µ_D_) between and within Kernza and annual wheat communities, as previously described [58]. As a measure of dissimilarity, a lower µ_D_ corresponds to more homogeneity (less changes or variations) in rhizomicrobiome composition on a scale of 0–1. In addition, stability of the rhizomicrobiome was estimated as mean ratio of change in relative abundance (∆RA) and standard deviation (s.d.) of ASVs between the 2 years, modified from a previous method [37]. We characterized the ‘core’ membership in Kernza and annual wheat rhizobacteria based on the ASV abundance-occupancy threshold of: ≥ 0.1% total reads and occurrence in ≥ 90% of samples separately for each crop type. Furthermore, we estimated the overlap between the endosphere and rhizosphere microbiomes based on the proportion (%) of shared ASVs in corresponding sample groups (Table S1). Since one rhizomicrobiome library corresponded to many endophyte libraries from the same sample source, we calculated the mean overlap for each paired group ($\overline{x}$_recruitment_). We also conducted a differential abundance (R package, *DESeq2, v. 1.42.1*) [59] analysis between the rhizosphere and endosphere microbiome communities of both crops to understand the rhizobacterial recruitment potential of both crops.

The AgroEcoDB is a curated database containing 16S rRNA gene amplicon libraries from 91 agroecosystem studies, encompassing a range of treatment factors related to soil disturbance (*ex*. tillage, drought, fertilization) and land management (*ex*. pasture, grassland, row cropping). It includes 71,440 ASVs annotated with environment-wide associations to these factors, along with corresponding indicator values for each treatment [46]. To evaluate whether ASVs associated with Kernza or annual wheat in our study reflected broader ecological patterns, we matched ASVs from our dataset to those annotated in AgroEcoDB which were indicators of land use (grassland associated ASVs), organic matter (organic soil horizon-associated), tillage regime (no-till-associated), and soil compartment (rhizosphere-associated). The relative abundances of ASVs matching each AgroEcoDB indicator category were aggregated, and differences in mean relative abundance between crops were tested using Wilcoxon rank-sum tests, with *P*-values adjusted for false discovery rate using the Benjamini–Hochberg procedure. All R code and data required to reproduce analyses are included as Supplementary Material which has been archived at the Open Science Framework (https://osf.io/) under the 10.17605/OSF.IO/K9U3F.

## Results

### Trends in alpha- and beta-diversity in the rhizomicrobiome of Kernza and annual wheat

**Fig. 1:**
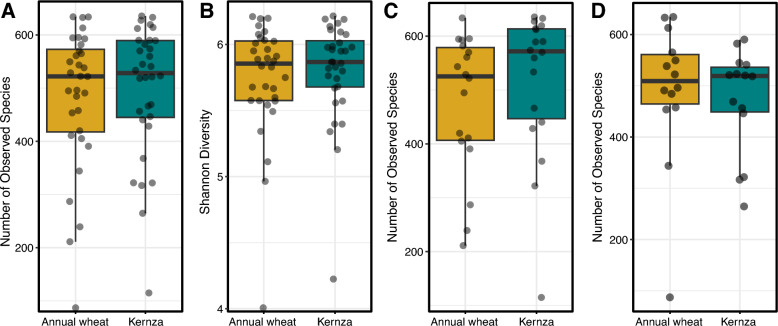
Alpha diversity in bacterial composition in the rhizosphere compared between annual wheat and Kernza aggregated (**A** and **B**) and at different depths (**C** = 5–15 cm and **D** = 25–35 cm)

**Fig. 2:**
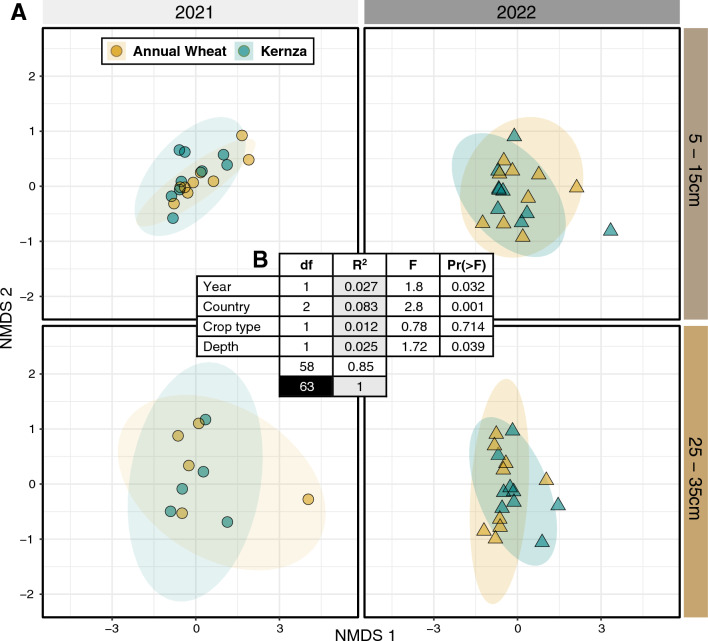
PERMANOVA analysis of effects of year, country, crop, and depth on rhizosphere bacterial community based on Bray–Curtis dissimilarity. Notes: community composition (**A**) and composition of rhizosphere bacterial community grouped by nonmetric multidimensional scaling (NMDS; stress = 0.1066) plot of sampling timepoints (year) and sampling depths (5–15 cm and 25–35 cm) (**B**). Circles and triangles represent years 2021 and 2022, respectively. Samples from all countries were merged

Overall, rhizobacterial populations of Kernza and annual wheat (‘crop type’) was highly similar across all sites, depths, and years. In contradiction of Hypothesis 1, rhizobacterial populations communities showed no significant difference in alpha-diversity (Shannon, Simpson, Chao1, or observed species) between the annual wheat and Kernza in aggregated (Fig. 1) as well as disaggregated data by study factors, namely sampling year, field site, and depth (Table S2). Whole rhizomicrobiome composition (beta-diversity) did not significantly differ by crop type as apparent in the high degree of clustering among Kernza and wheat rhizomicrobiomes, regardless of year or depth (Fig. 2A). According to PERMANOVA, the agro-climate zone (or country) explained the greatest variation in rhizomicrobiome composition (R^2^ = 0.083) (Fig. 2B), with year (0.027), and depth (0.025) also showing smaller, but significant, impacts on rhizomicrobiome composition (Fig. 2B).

### Crop-associated and core rhizobacteria, and endophytes of annual wheat and Kernza

**Fig. 3:**
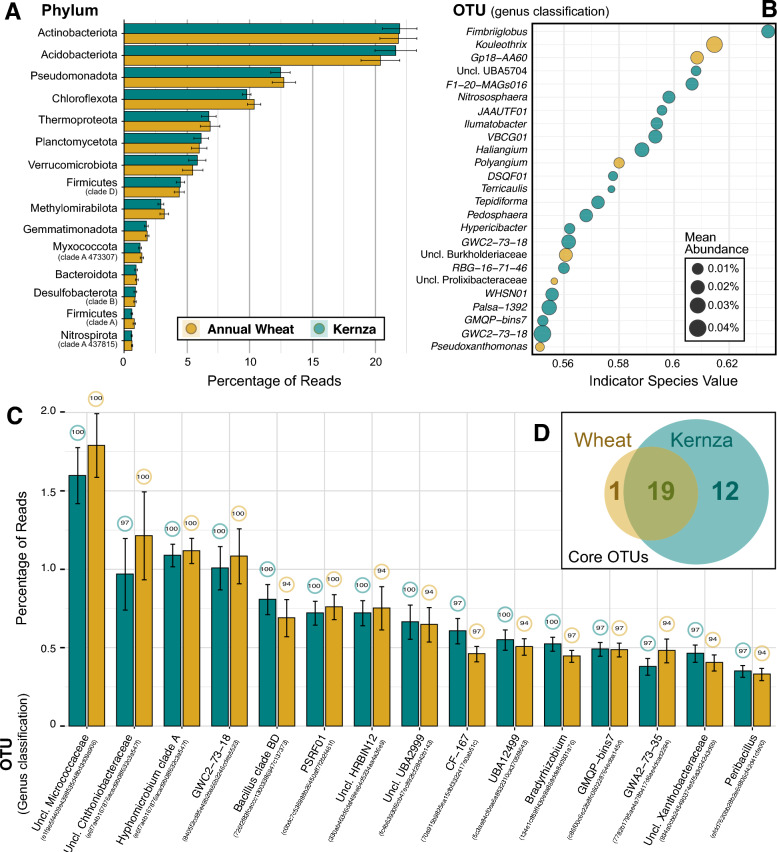
Comparison of phyla, core ASVs, and indictor ASVs between both crops. Notes: most abundant phyla between both crops (**A**), most abundant crop-associated ASVs visualized at indicator value threshold of ≥ 5.5 (**B**), and most abundant core ASVs common between both crop types visualized at a threshold of ≥ 0.91 occupancy. Values on top of the error bars represent occupancy (**C**). Error bars indicate standard error of the relative abundance. In (**D**), a Venn diagram showing the number of ‘core’ ASVs identified in each crop and the overlap among crops

We used several approaches to compare trends that differed between the rhizobacteria of annual wheat and Kernza. Broadly, at the phylum rank, the rhizobacteria between Kernza and annual wheat were largely similar. The relative abundance of the most prevalent phyla (≥ 1%) were at parity between the two crops, with no statistical differences for any of the most abundant phyla based on Wilcoxon test (Fig. 3A). The most dominant bacterial phyla (≥ 10%) in the rhizobacteria were Actinobacteriota (${\overline{\text{x}}}_{{_{{{\text{wheat}}}} }}$  = 21.6% and ${\overline{\text{x}}}_{{_{{{\text{Kernza}}}} }}$  = 22.2%), Acidobacteriota (${\overline{\text{x}}}_{{_{{{\text{wheat}}}} }}$  = 21.8% and ${\overline{\text{x}}}_{{_{{{\text{Kernza}}}} }}$  = 20.3%), Pseudomonadota (${\overline{\text{x}}}_{{_{{{\text{wheat}}}} }}$  = 13%, ${\overline{\text{x}}}_{{_{{{\text{Kernza}}}} }}$  = 12%), and Chloroflexota (${\overline{\text{x}}}_{{_{{{\text{wheat}}}} }}$  = 9.8% and ${\overline{\text{x}}}_{{_{{{\text{Kernza}}}} }}$  = 10.4%), out of the 72 total phyla observed (list of all observed phyla; Table S3). Two archaeal phyla were also detected: Thermoproteota (${\overline{\text{x}}}_{{_{{{\text{wheat}}}} }}$  = 7% and ${\overline{\text{x}}}_{{_{{{\text{Kernza}}}} }}$  = 7%) and Methylomirabilota (${\overline{\text{x}}}_{{_{{{\text{wheat}}}} }}$  = 2.8% and ${\overline{\text{x}}}_{{_{{{\text{Kernza}}}} }}$  = 3.3%), and also did not differ by crop type.

Despite the overarching similarities in rhizobacteria between crops, we identified differentially abundant ASVs associated with either Kernza (n_ASV_ = 115) or annual wheat (n_ASV_ = 54) using indicator species analysis (Fig. 3B; full list in Table S4). At the phylum level, many Kernza and wheat-associated ASVs were classified to Planctomycetota and Chloroflexota and twelve genera were commonly associated with both crops. Notably, Kernza-associated ASVs were members of a diverse 51 genera, including *Fimbriiglobus* and *Haliangium*, while wheat-associated ASVs were members of 22 genera, including *Kouleothrix* and *Nocardioides* clade A.

To identify microbial taxa consistently associated with each crop, we independently examined the core rhizobacteria of Kernza, then annual wheat, based on occupancy (≥ 90% of samples) and relative abundance (≥ 0.1%) thresholds. A total of 31 and 20 ASVs met the criteria of core membership in Kernza or annual wheat, respectively (complete list in Table S5). A total of 19/51 ASVs were core to the rhizobacteria of both crops (Fig. 3D), meaning they met the threshold for all samples (Fig. 3C). Among the core taxa of Kernza, the most prevalent genera (100% occupancy) included *Bradyrhizobium*, *Hyphomicrobium* clade A and *Bacillus* clade BD while annual wheat had *Hyphomicrobium* clade *A* as the most prevalent genus.

### The influence of crop type on rhizomicrobiome composition at depth

**Fig. 4:**
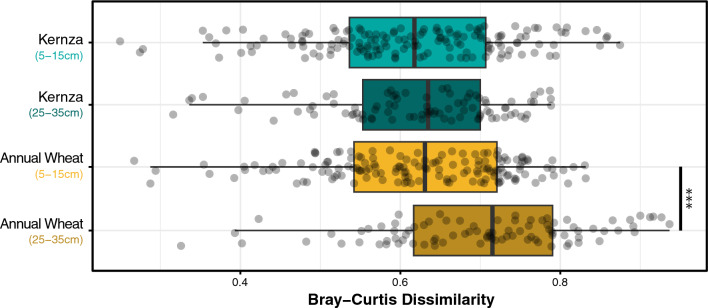
Comparison of rhizomicrobiome changes (dissimilarity) between and within crop types (Kernza and annual wheat) at depth. Significance annotations *, **, ***, ns represent *P* < 0.01, 0.001, 0.0001, and > 0.05, respectively based on Wilcoxon test

We evaluated two key trends in rhizomicrobiome community structure to test whether perennialization influences populations differently with soil depth, based on the expectation that Kernza has a more extensive root system (Hypothesis 2). First, we compared whether the rhizomicrobiome was more homogeneous at different depths in each of the crop types (which included variation among sites and across years). We observed no difference in the Bray–Curtis mean dissimilarity (µ_D_) between Kernza rhizomicrobiomes at 5–15 cm (µ_D_ = 0.61) and 25–35 cm depths (µ_D_ = 0.62; *P*_*adj*_ < 0.98), meaning that, on average, any two communities sampled at either depth were generally similar (Fig. 4). Conversely, we observed significantly higher µ_D_ between rhizomicrobiomes in annual wheat at the lower depth (µ_D_ = 0.71; *P*_*adj*_ < 0.001) than in surface soil (µ_D_ = 0.62), reflecting a greater heterogeneity among rhizomicrobiome communities at depth. Our observation support that, on deeper soils, Kernza rhizomicrobiomes were significantly more homogeneous (lower µ_D_) than annual wheat.

**Fig. 5:**
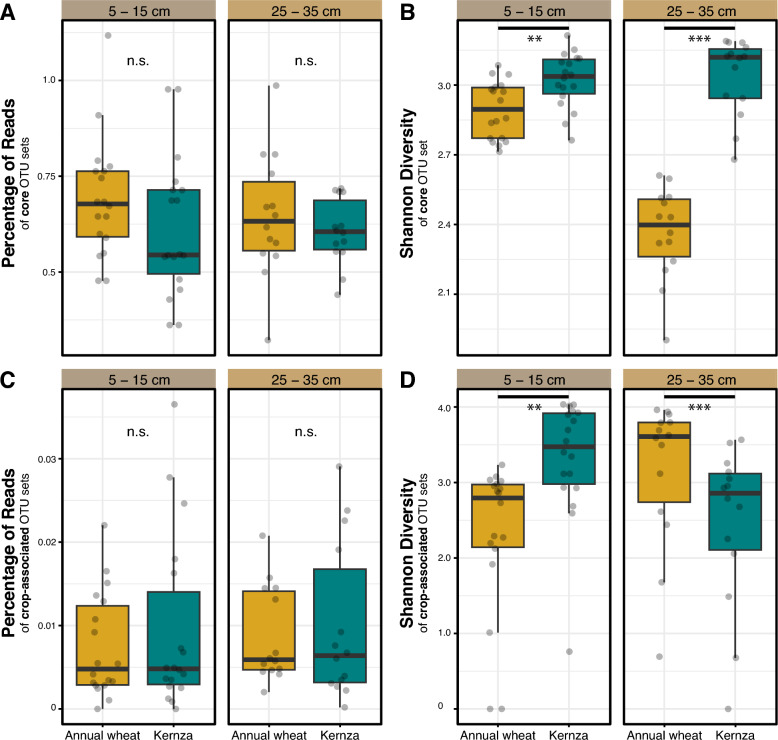
Relative abundance (**A**) and diversity (**B**) of core ASVs, and relative abundance (**C**) and diversity (**D**) of crop-associated ASVs compared at depth. Significance annotations *, **, ***, ns represent *P* < 0.01, 0.001, 0.0001, and > 0.05 respectively based on Wilcoxon test

Next, we determined whether depth-dependent differences were driven by crop-associated or core populations. No significant differences were observed in the relative abundance of the core ASVs between the depths 5–15 cm and 25–35 cm of Kernza (Wilcoxon; *P*_*adj*_ = 0.66) or annual wheat (*P*_*adj*_ = 0.66). When compared between annual wheat and Kernza at depths, there was no significant difference (5-15 cm, *P*_*adj*_ = 0.38; 25–35 cm, *P*_*adj*_ = 0.57) in the relative abundance of core ASVs between both crops (Fig. 5A; Table S6). Nonetheless, the core ASVs in Kernza exhibited a significantly higher Shannon diversity (*P* < 0.001) compared to wheat at both depths (Fig. 5B; Table S6), with a more pronounced difference at depth. Crop-associated ASVs—both between and within crop types —revealed no significant difference (*P*_*adj*_ = 0.87) at depth in the relative abundance of the crop-associated rhizomicrobiome (Fig. 5C, Table S6). However, Kernza mostly had a higher (*P*_*adj*_ < 0.001) diversity compared to annual wheat at the upper soil depth, while the opposite is true for annual wheat (*P*_*adj*_ = 0.019) at lower depth (Fig. 5D).

### The influence of crop type on rhizomicrobiome stability over time

**Fig. 6:**
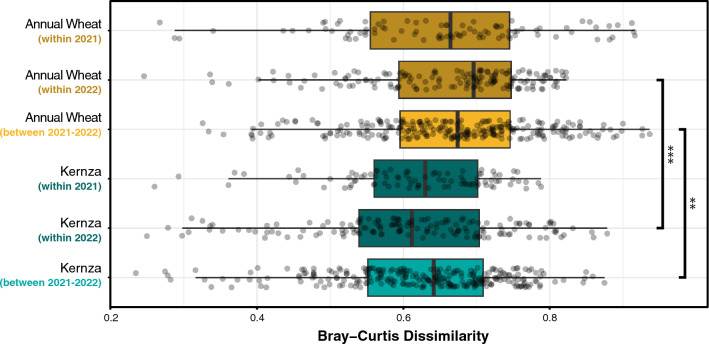
Year-on-year comparison in rhizomicrobiome changes (dissimilarity) between and within crop types (Kernza and annual wheat). Significance annotations *, **, ***, ns represent *P* < 0.01, 0.001, 0.0001, and > 0.05, respectively based on Wilcoxon test

We tested whether perennialization reduced year-to-year variation in rhizobacterial communities, based on the continuous presence of Kernza’s permanent root system (Hypothesis 3). First, we compared the µ_D_ among rhizobacterial communities within and between years for both Kernza and annual wheat. We did not observe any significant difference in µ_D_ within a year or between years for each separate crop (Fig. 6), indicating that rhizobacterial communities with the same crop type were, on average, equally similar regardless of whether samples were taken in the same or different years. Notably, the between-year µ_D_ of Kernza (µ_D_ = 0.62) was significantly lower than for annual wheat (µ_D_ = 0.67; *P*_*adj*_ = 0.001), indicating that the Kernza rhizomicrobiome was, on average, more similar across years than annual wheat. Annual wheat exhibited a significantly higher heterogeneity than Kernza in both years (*P*_*adj*_ < 0.0017), suggesting that the rhizomicrobiome composition was on-the-whole more variable than Kernza. Additionally, we determined the year-on-year stability in the relative abundance (∆_RA_) and standard deviation (s.d.) of individual ASVs (∆_RA_ of individual ASVs analyzed; Table S7). We observed that ASVs in the Kernza rhizomicrobiomes (∆_RA_ = 0.89, s.d. = 2.21) were, on average, 12.0% lower than annual wheat (∆_R.A._ = 1.01, s.d. = 2.38). This difference was significant (*P* < 0.0001), indicating the rhizomicrobiome of Kernza, on average, remained more stable than annual wheat between the 2 years (Fig. S1).

### The influence of crop type on the recruitment of root endophyte populations

We analyzed the overlap between the root endophytes and rhizomicrobiome to understand the influence of perennialization on the recruitment of endophytes from the rhizosphere. The alpha- and beta-diversity of rhizomicrobiome and endophyte communities broadly differed in both annual wheat and Kernza, a common observation between these two crop compartments. Endophytes communities had significantly lower alpha-diversity than rhizobacterial communities for both crops (*P* < 0.001) based on multiple metrics: Chao1, Shannon, Simpson, and observed species (Fig. S2, annual wheat; Fig. S3, Kernza). An NMDS ordination, based on Bray–Curtis dissimilarity, confirmed that rhizobacterial populations and endophytes were distinct in composition and illustrated a greater variation in endophyte community composition (Fig. S4).

**Fig. 7:**
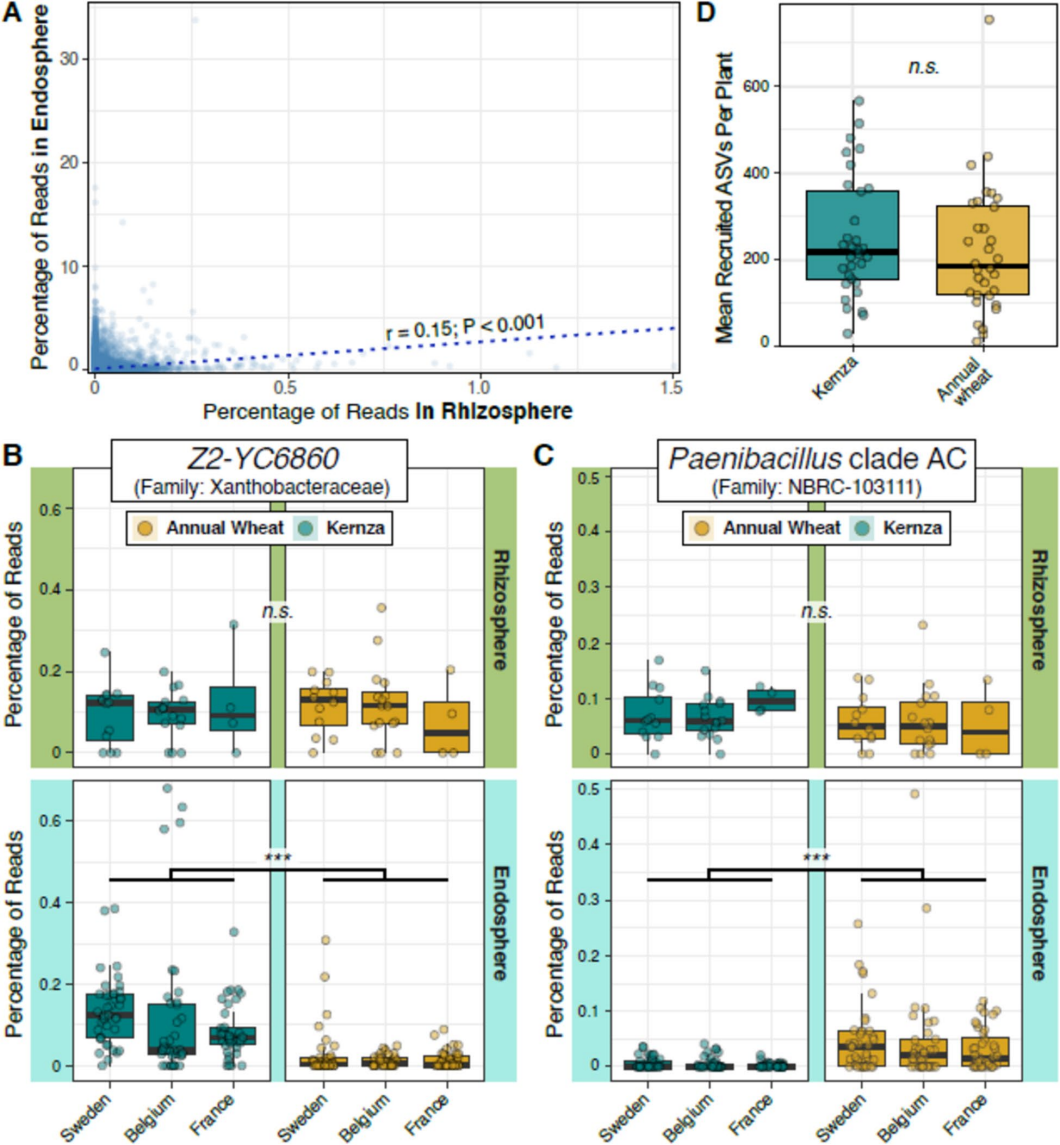
Comparison of rhizomicrobiome recruitment potential of Kernza and annual wheat. Notes: overarching correlation between the endosphere and rhizosphere microbiome for both crops (**A**), ASVs showing notable patterns in the endosphere and rhizosphere of Kernza and annual wheat (**B** and **C**). The percentage of reads reflects the aggregated value for ASVs that showed the same individual pattern. Panel **D** is the mean recruitment of ASVs based on paired comparisons. Significance annotations *, **, ***, ns represent *P* < 0.01, 0.001, 0.0001, and > 0.05, respectively based on Wilcoxon test. Endophyte data used were obtained from Michl et al. [45]

A total of 3,667 ASVs were differentially abundant in the endosphere relative to the rhizosphere in both crops, of which 13.4% (n_ASV_ = 491) were consistently identified as endophytes in all three countries (Table S8). There was no significant difference in the relative abundance of endophytes between Kernza and annual wheat, whether the whole set (Wilcoxon test; *P* = 0.16), or country-wide subset (Wilcoxon test; *P* = 0.86) were tested. A total of 274 ASVs (55.8%) identified as endophytes were present in the rhizosphere, at an average abundance of 0.7%. The correlation between the relative abundance of root endophyte ASVs and their abundance in the rhizosphere was weak (*r* = 0.15, *P* < 0.001; Fig. 7A), but slightly stronger than the correlation observed when considering all ASVs (including non-endophyte ASVs) in the dataset (*r* = 0.04, *P* < 0.001). Among endophytes detected in all three countries, 29 ASVs were differentially abundant in Kernza and 9 in annual wheat roots (Table S9). Most of these ASVs were absent or in very low abundance in the rhizobacterial community (*ex. Herpetosiphon* in annual wheat). However, two genera exhibited notable patterns in relative abundance, occurring at similar relative abundances in the rhizosphere of both crops but differentially abundant in the endosphere of only one crop, suggesting preferential recruitment. These included *Z2-YC6860* (Xanthobacteraceae) for Kernza and *Paenibacillus* clade AC in annual wheat (Fig. 7BC).

The recruitment of endophytes from rhizobacterial communities based on the degree of overlap (i.e., presence in rhizosphere and root endosphere) did not reveal striking differences between crops. Kernza had a total of 2,086 unique overlapping ASVs, which was a slightly higher proportion (29.5%) than annual wheat, which recruited a total of 1,954 rhizobacterial ASVs (27.6%), with the remaining 42.9% common to both crops. There was no statistically significant difference in the number of recruited ASVs between Kernza (mean = 247 ASVs) and annual wheat (mean = 219 ASVs) across paired samples (*P* = 0.28; Fig. 7D).Differential abundance analysis of recruited ASVs common for both crop types, revealed that Kernza (n_ASV_ = 434) had a higher share of differentially abundant ASVs with a significant (Wilcoxon test; *P*_*adj*_ < 0.0001) mean abundance than annual wheat (n_ASV_ = 109) (Fig. S5).

### Environment-wide associations of Kernza- and annual wheat-associated populations

**Fig. 8:**
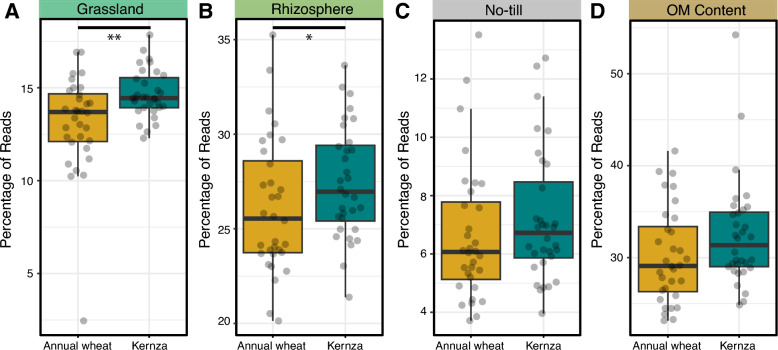
Comparison of rhizosphere microbiome of Kernza and annual wheat with agroecology database. Factors: **A **grassland; **B** rhizosphere; **C** no till; and **D** organic matter soil. Significance annotations *, **, ***, ns represent *P* < 0.01, 0.001, 0.0001, and > 0.05, respectively based on Wilcoxon test

We mapped ASVs from the Kernza and annual wheat rhizobacteria to the AgroEcoDB to test for associations with grassland soil microbiomes, other crop rhizobacteria, fields where no-till is practiced, or where organic-rich soil was present. A total of 7019 out of 20,074 ASVs (35%) from Kernza and annual wheat rhizobacteria matched ASVs in the AgroEcoDB. In aggregate, rhizobacteria populations with environment-wide associations with grassland soils (n_ASV_ = 442) and rhizosphere soils (n_ASV_ = 1034) were significantly more abundant in the rhizobacteria of Kernza than annual wheat (Fig. 8AB). There were 33 grassland-associated ASVs previously identified as indicators of Kernza driving this trend, with the most prevalent ASV classified to *Haliangium* (Phylum: *Myxococcota*, “820e119abd5d01b8fb1880bdcd5562ab”). An ASV classified as *Bacillus* clade BD (Phylum: Firmicutes clade D, “726f2f83fcaccc13003386947c137373”) drove the difference in relative abundance of rhizosphere-associated ASVs between crops. This ASV was previously identified as a prevalent core member of the Kernza microbiome, but not of annual wheat. ASVs attributed to no-till (n_ASV_ = 166, Fig. 8C) and organic matter concentration (n_ASV_ = 634, Fig. 8D) did not exhibit significant differences in relative abundance between Kernza and annual wheat rhizobacteria.

## Discussion

Our study assessed the influence of perennialization through Kernza cultivation (*T. intermedium*) on the structure and stability of rhizobacterial populations, and source of endophyte populations, compared to annual wheat (*T. aestivum*). We tested four hypotheses: (1) Kernza would facilitate higher alpha-diversity in rhizobacterial populations; (2) Kernza would exhibit a more pronounced rhizomicrobiome at deeper soil depths due to deep-rooting behavior; (3) Kernza would demonstrate lower variation in beta-diversity from year to year, indicating a more stable microbial community; and (4) Kernza would recruit a higher proportion of endophyte populations from rhizobacterial populations than annual wheat. Overall, our findings show that country (representing different agroclimatic and soil conditions), soil depth, and sampling year exerted a greater influence than the selective pressure of crop type on rhizomicrobiome composition (Fig. 2). The general lack of differences may be attributed to the shared genetic ancestry of Kernza and annual wheat [60], potentially leading to similar host genetic traits for recruiting microbial populations [61, 62], or environmental factors may dominate the crop effect, as agroclimatic conditions often play a dominant role in shaping microbial communities [63]. While rhizomicrobiome composition did not differ substantially between these two cropping systems after 4–6 years, we observed notable differences that provide nuanced insights into our original hypotheses, which we discuss in detail below.

### Higher alpha-diversity in Kernza rhizomicrobiome (Hypothesis 1)

Contrary to our first hypothesis, no significant differences in the alpha-diversity of rhizobacterial populations were observed between Kernza and annual wheat across all study factors: country, depth, and time (Fig. 1). This lack of difference stands in contrast to the root endophyte populations from the same samples, which showed clear differences in richness [45]. The stronger influence on species richness and evenness in the endosphere compared to the rhizosphere suggests that host-specific factors, such as cell wall composition, immune responses, or root metabolite profiles [64], play a stronger role than the cultivation of a diverse rhizosphere. To this point, many endophyte ASVs were absent or occurred at very low abundance in the rhizosphere (Fig. 7A). Patterns in alpha-diversity are influenced by different community assembly processes and can fluctuate over short timescales [65]. However, in more stable systems, alpha-diversity often increases over time as communities mature and ecological niches diversify [66, 67]. Thus, our findings reflect the state of communities after 4–6 years, and any differences in species richness or evenness in rhizobacterial communities between crops may take longer to emerge. Our results are consistent with another long-term study (3 years) comparing Kernza and annual wheat systems reported no differences in rhizobacterial alpha-diversity, but differences in rhizosphere-dwelling fungi [42]. Notably, Sprunger et al. [40] found that rhizobacterial and nematode alpha-diversity had changed, but only after four years of Kernza establishment, illustrating the context-dependent nature of assessing species richness and evenness. Therefore, while Kernza offers numerous ecological benefits, effects on rhizobacterial alpha-diversity may not be among its primary effects. This conclusion requires more research for confirmation, as studies are still limited.

### Influence of rooting depth on rhizomicrobiome (Hypothesis 2)

Our results revealed several insights into how the perennial, deeper-rooting growth habit of Kernza influences rhizobacterial communities. Firstly, community heterogeneity (µ_D_) within rhizobacterial populations of annual wheat was greater than Kernza in deeper soils (Fig. 4). Since roots tend to exert a homogenizing influence on microbial communities (exudation, immune response etc.), an increase in variation of beta-diversity in deeper annual wheat rhizosphere may be interpreted as a weakening of root influence [68, 69]. From this, we conclude that the increased heterogeneity in annual wheat rhizomicrobiome at 25–35 cm likely reflects the differences in root mass in these samples. Where fewer, smaller roots exist, the recovery of rhizosphere soil via coring is subject to a greater dilution from bulk soil communities, corresponding to the higher heterogeneity in composition we observed in annual wheat. Our observation suggests differences in root architecture and rhizodeposition may be revealed through signatures in rhizomicrobiome data, as previously noted [16, 70, 71], though confirming these findings will require the collection of root mass data, which we do not possess. The environment-wide analysis highlighted that rhizosphere-associated rhizobacterial populations occupied a significantly higher proportion of the rhizobacterial community in Kernza than annual wheat, further supporting a stronger influence of Kernza on the rhizomicrobiome.

Depth did not exert a strong or pronounced effect on the relative abundance of crop-associated and/or core members of either Kernza or annual wheat rhizobacterial communities, except in terms of alpha-diversity trends which are difficult to interpret (Fig. 5). These observations raise questions about the nature of the influence of Kernza at depth, since a stronger selection due to root mass and/or rhizodeposition (i.e., resource availability) would be expected to influence the relative abundance of Kernza associated taxa. Regarding the trend in alpha-diversity, prior research in soybean reported a similar trend, with increasing rhizobacterial alpha-diversity at increasing root depth in soybean [*Glycine max* (L.) Merrill] [70]. Root age, inferred from plant age, may be one factor underlying these observations, where young roots, which may be more abundant in deeper soils, are more active sources of exudates, thus recruiting more of the core/crop-associated rhizobacterial taxa [63]. These interpretations remain speculative and require further research that compares the root mass, root exudation, and community assembly of each crop across depth.

### Increase inter-annual stability in rhizomicrobiome of Kernza (Hypothesis 3)

The perennial root system of Kernza provides a continuous habitat and resource supply for rhizomicrobiome populations, potentially reducing fluctuations in community composition between growing seasons. Our observations indicated that Kernza rhizobacterial populations were more stable than annual wheat with ~ 11% less interannual variation. Kernza also maintained a slightly higher number of core ASVs, a feature previously associated with more stable trajectories of rhizosphere communities [72], and harbored a greater relative abundance of ASVs associated with grassland soils, according to the AgroEcoDB. This pattern was consistent across all countries and depths, suggesting a stronger ecological semblance between Kernza fields and the rhizospheres of undisturbed grasslands [19, 73].

In support of this view, one of the most differentially abundant grassland-associated ASV in Kernza was classified as *Haliangium*, a genus known to contain predatory bacteria [74, 75] and previously found to be enriched in *T. intermedium* [22]. A higher prevalence of *Haliangium* may indicate a more developed microbial trophic network in the Kernza rhizosphere, consistent with the role of bacterial predators [76]. Prior reports of complex microbial food webs in Kernza fields [40], strengthen this interpretation. Regardless, *Haliangium* are frequently reported in microbiome surveys of grasslands (in studies absent from the AgroEcoDB) and have been identified as a keystone taxon during the reclamation of agricultural fields to grasslands [74, 75, 77].

The enhanced stability observed in Kernza may reflect crop traits, such as root longevity and C inputs [78], and a K-selected growth strategy that favors investment in microbial associations [28, 36]. The stability conferred by these Kernza traits is ecologically advantageous, as the maintenance of consistent microbial populations can lead to resilience to disturbances [79] and offers potential targets for breeding programs. For future breeding approaches, it would be valuable to identify Kernza-specific genetic traits that are involved in shaping the microbiome, so-called Microbiome (M) genes [80]. Knowledge about such genes might even be useful to improve annual cereals via introgression of desirable M genes.

### Stronger recruitment and internalization of endophyte populations (Hypothesis 4)

Our fourth hypothesis proposed that Kernza would recruit a greater proportion of its endophyte community from rhizobacterial populations than annual wheat, owing to its more enduring root influence. Overall, the correlation between ASV relative abundances in the rhizosphere and endosphere was very weak (Fig. 7A), suggesting that fitness in the rhizosphere is a poor predictor of colonization success. This weak correlation may reflect the substantial contribution of endophytes originating from the seed microbiome or from early-colonizing populations that declined over time.

Using the broadest definition of recruitment (i.e., simple overlap in ASV membership between the rhizosphere and endosphere), Kernza did recruit a slightly higher total number of ASVs. However, when comparing the mean number of recruited ASVs at the field scale, there were no differences between crop types (Fig. 7B). This lack of broad differentiation suggests that both crops are similarly permissive in their recruitment from the rhizosphere, with approximately 56% of endophytes found in all three countries also present in the rhizosphere, the other half likely present in the seed or at low rhizosphere abundance.

Only a small subset of recruited taxa (n_ASV_ = 38) was differentially associated with one crop, the majority of which (76%) were associated with Kernza. These findings suggest that while Kernza may indeed recruit a slightly greater proportion of its endophyte community from rhizobacterial sources, this effect appears subtle and occurs at a fine scale, rather than representing a dominant pattern. Among these, were at least two rhizobacterial groups that illustrated preferential recruitment by Kernza and annual wheat (Fig. 7B, C), illustrating difference in the recruitment potential of both crops, likely due to root structures (*ex*. intercellular root spaces) or recruitment mechanisms [16, 81]. Understanding the differences in recruitment mechanisms and source of endophytes in Kernza, along with their influence on plant fitness, can inform the ongoing domestication of Kernza. This is particularly important when one considers that breeding cycles have had an inverse effect on the evenness and richness of seed endophytes of Kernza [82]. Rhizomicrobiome recruitment can be a strategy for stress mitigation and improved nutrient acquisition [69]. For example, the top Kernza-associated ASV belong to the genus *Fimbriiglobus* (Phylum: Planctomycetes, “8ac560af4648e5083d4f5afb14420619”), a poorly understood bacterial clade that is known to degrade chitin, and perform rock weathering [83–85]. The small but distinct subset of endophytes preferentially recruited by Kernza warrants closer investigation, as uncovering their functional roles could reveal how perennial crops selectively engage microbial partners to enhance nutrient cycling, stress tolerance, or long-term plant health [86].

### Implications for agricultural practices and the domestication of Kernza

The influence of plant genotype on rhizomicrobiome structure and function is well established and, in some cases, has been quantitatively mapped to specific plant loci [63, 87]. In the case of wheat, domestication resulted in minor, but consistent, differences in genotypic variation for the selection of a rhizomicrobiome, when comparing modern cultivars and wild wheat [88]. Increasing attention is being paid to how breeding programs, like the domestication of Kernza, may incorporate rhizomicrobiome traits into breeding targets [89, 90]. Our findings show that the Kernza and annual wheat exert comparable rhizosphere influences at a higher taxonomic scale, but at a microdiversity level, Kernza showed greater influence on the rhizomicrobiome. The seeming lack of divergent selection, also reported elsewhere [42], suggests that long-term selection for grain yield, and other traits of domestication, did not have far reaching consequences on rhizobacterial community structure. Notwithstanding, available evidence suggests that breeding selection has had a significant influence (mostly negative) on the alpha diversity, composition, and abundance of seed endophytes across breeding cycles of Kernza [82]. Future efforts to distinguish if, and why, conditions in the rhizosphere of wheat, and related intermediate grasses, are insensitive to domestication may yield insights into paths for breeding without altering rhizomicrobiome selection. Additionally, a determination should be made of the ecology and function of the divergent crop-associated populations to understand if the minor changes in composition have outsized impact on rhizomicrobiome function. Notably, the broad similarities in rhizobacterial populations suggest that succeeding crops may benefit equally from either crop (Kernza or annual wheat) regarding the agroecological function of their associated microbiomes. This remains speculative until the overall effect of the notable differences at the microdiversity scale on the rhizomicrobiome between both crops is verified.

## Conclusions

This study found modest differences in rhizomicrobiome composition between Kernza and annual wheat, with Kernza positively influencing the rhizomicrobiome overall—promoting rhizomicrobiome stability, higher recruitment of endophytes, and hosting significant proportions of grassland-associated as well as rhizosphere-dwelling microbiomes. We conclude that rhizomicrobiome composition of Kernza and annual wheat are mostly similar—with differences attributed to depth, year (growth stage), and country (agro-climatic condition). But at the microdiversity scale, Kernza exerted influence on the rhizomicrobiome. Thus, while Kernza may have significant positive effects in enhancing biodiversity as reported for other organisms, this effect seems less pronounced on rhizobacterial communities. Nonetheless, Kernza possesses a potential to ensure stability of rhizomicrobiome over time. Such a function suggests a minimal disruption of the ecosystem which is beneficial for ecosystem health and protection of important habitats. Furthermore, Kernza recruits differentially abundant ASVs from the rhizomicrobiome for internalization. Our results also highlight the fact that Kernza hosts a higher proportion of grassland-associated and rhizosphere-associated taxa than annual wheat—providing important evidence for the influence of disturbance on rhizobacterial communities. While previous reports have suggested that Kernza mimics natural grasslands [7, 28], our data suggest the potential for Kernza to provide similar microbially-mediated ecosystem services as natural grasslands. Future research may benefit our understanding of these services by investigating the influence of Kernza at depth, and the mechanisms by which Kernza recruits its microbiome, including the potential role of plant-derived metabolites in shaping community assembly.

## Supplementary Information

Supplementary Material 1

Supplementary Material 2

Supplementary Material 3

Supplementary Material 4

Supplementary Material 5

Supplementary Material 6

Supplementary Material 7

Supplementary Material 8

Supplementary Material 9

Supplementary Material 1

Supplementary Material 11

Supplementary Material 12

Supplementary Material 13

Supplementary Material 14

## Data Availability

Data from this study is shared in the supplementary material and amplicon sequencing data for root endophytes has been deposited in the NCBI Sequence Read Archive under the BioProject accession number: PRJEB74910, while the rhizosphere amplicon sequencing data for rhizosphere has been deposited on Open Science Framework (https://osf.io/) under the 10.17605/OSF.IO/K9U3F.
