# Supplementary material for: Perennial Kernza cropping promotes rhizosphere microbiome stability and endophyte recruitment compared to annual wheat
Source: Environ Microbiome. 2025 Nov 7;20:139. doi: 10.1186/s40793-025-00794-3 (PMC12595868; doi:10.1186/s40793-025-00794-3)
Supplement: Supplementary file 1 — Supplementary Material 1 [file 40793_2025_794_MOESM1_ESM.pdf]

Perennial Kernza Cropping Promotes Rhizosphere Microbiome Stability and Endophyte Recruitment Compared to Annual Wheat

Sulemana Issifu[1,2], Arval Viji Elango[2], Kristina Michl[3], Christophe David[4], Tomislav Cernava[3,5], Roland C. Wilhelm[2], and Frank Rasche[1,6]

[1]Institute of Agricultural Sciences in the Tropics (Hans-Ruthenberg-Institute), University of Hohenheim, Garbenstr. 13, 70599, Stuttgart, Germany.

[2]Department of Agronomy, Lilly Hall of Life Sciences, Purdue University, USA

[3]Institute of Environmental Biotechnology, Graz University of Technology, Graz 8010, Austria

[4]Department of Agroecosystems, Environment and Production, ISARA, Lyon Cedex 07, France

[5]School of Biological Sciences, Faculty of Environmental and Life Sciences, University of Southampton, SO171BJ Southampton, United Kingdom

[6]Present address: International Institute of Tropical Agriculture (IITA), P.O.

Box 30772-00100, Nairobi, Kenya

Description: Figure S1 is a comparison of the cumulative ratio of change in relative abundance of ASV between annual wheat for two years (2021 and 2022).

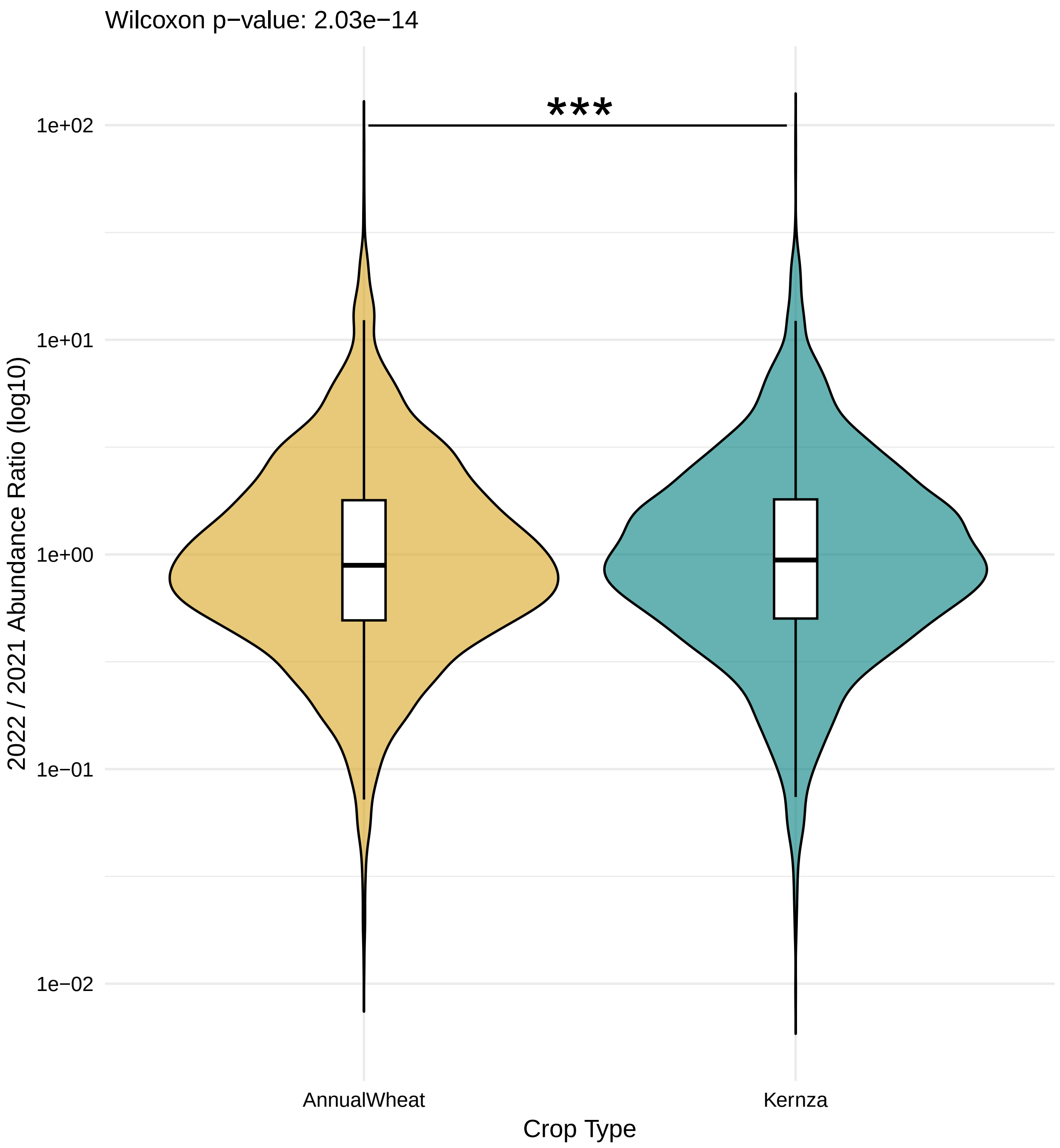

Wilcoxon p−value: 2.03e−14
***
1e+02
1e+01
1e+00
1e−01
1e−02
2022 / 2021 Abundance Ratio (log10)
AnnualWheat
Kernza
Crop Type
