# Supplementary material for: Perennial Kernza cropping promotes rhizosphere microbiome stability and endophyte recruitment compared to annual wheat
Source: Environ Microbiome. 2025 Nov 7;20:139. doi: 10.1186/s40793-025-00794-3 (PMC12595868; doi:10.1186/s40793-025-00794-3)
Supplement: Supplementary file 2 — Supplementary Material 2 [file 40793_2025_794_MOESM2_ESM.pdf]

Perennial Kernza Cropping Promotes Rhizosphere Microbiome Stability and Endophyte Recruitment Compared to Annual Wheat

Sulemana Issifu1,2, Arval Viji Elango2, Kristina Michl3, Christophe David4, Tomislav Cernava3,5, Roland C. Wilhelm2, and Frank Rasche1,6

1Institute of Agricultural Sciences in the Tropics (Hans-Ruthenberg-Institute), University of Hohenheim, Garbenstr. 13, 70599, Stuttgart, Germany.

2Department of Agronomy, Lilly Hall of Life Sciences, Purdue University, USA

3Institute of Environmental Biotechnology, Graz University of Technology, Graz 8010, Austria

4Department of Agroecosystems, Environment and Production, ISARA, Lyon Cedex 07, France

5School of Biological Sciences, Faculty of Environmental and Life Sciences, University of Southampton, SO171BJ Southampton, United Kingdom

6Present address: International Institute of Tropical Agriculture (IITA), P.O. Box 30772-00100, Nairobi, Kenya

* Corresponding Author: Frank Rasche (f.rasche@cgiar.org)

Description: Figure S2 is a comparison of alpha diversity between the rhizosphere and root endosphere of annual wheat based on multiple metrices.

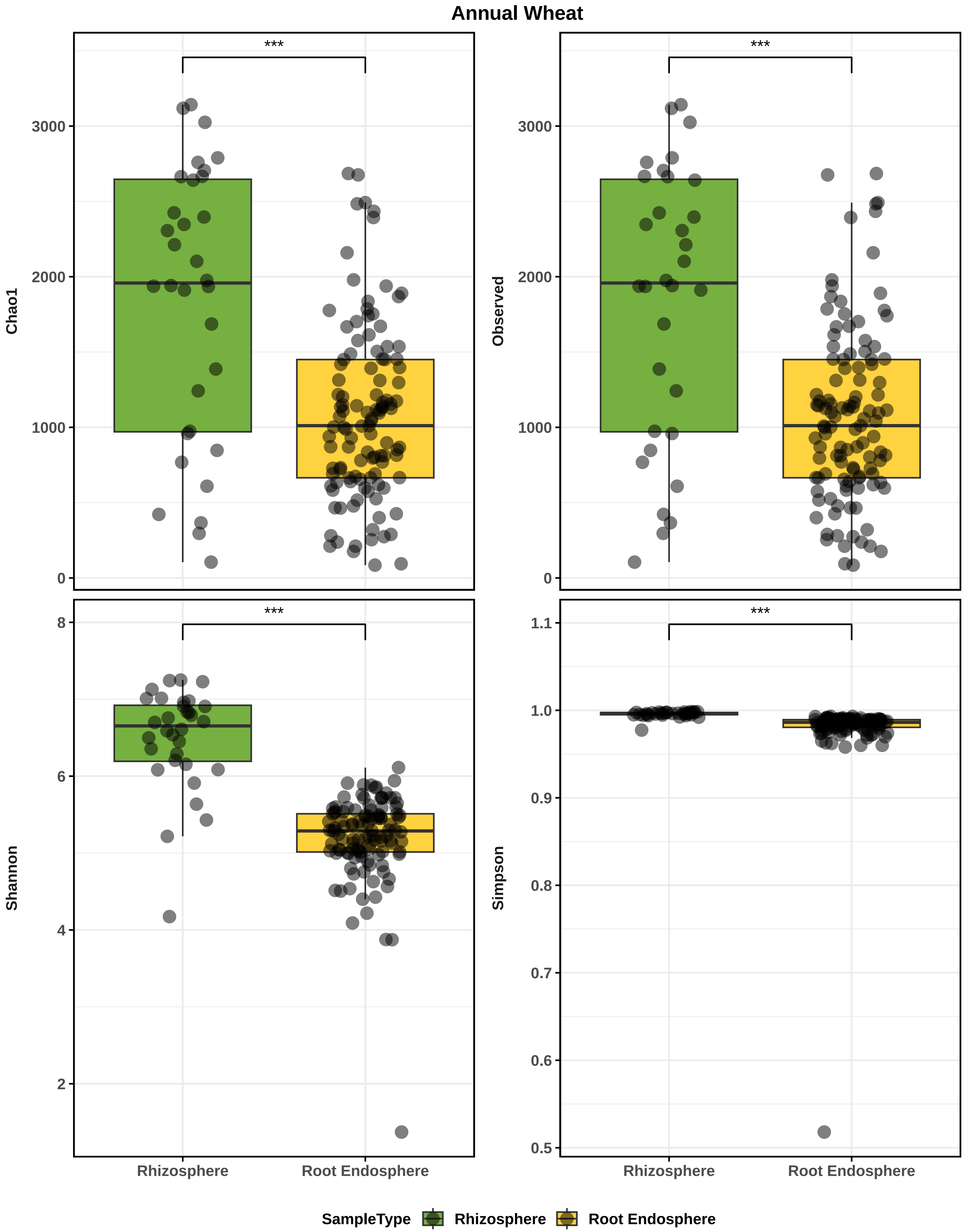

Annual Wheat
***
Chao1
3000
2000
1000
0
Observed
Shannon
8
6
4
2
Simpson
1.1
1.0
0.9
0.8
0.7
0.6
0.5
Rhizosphere
Root Endosphere
SampleType
