# Supplementary material for: Perennial Kernza cropping promotes rhizosphere microbiome stability and endophyte recruitment compared to annual wheat
Source: Environ Microbiome. 2025 Nov 7;20:139. doi: 10.1186/s40793-025-00794-3 (PMC12595868; doi:10.1186/s40793-025-00794-3)
Supplement: Supplementary file 5 — Supplementary Material 5 [file 40793_2025_794_MOESM5_ESM.pdf]

Perennial Kernza Cropping Promotes Rhizosphere Microbiome Stability and Endophyte Recruitment Compared to Annual Wheat

Sulemana Issifu[1,2], Arval Viji Elango[2], Kristina Michl[3], Christophe David[4], Tomislav Cernava[3,5], Roland C. Wilhelm[2], and Frank Rasche[1,6]

[1]Institute of Agricultural Sciences in the Tropics (Hans-Ruthenberg-Institute), University of Hohenheim, Garbenstr. 13, 70599, Stuttgart, Germany.

[2]Department of Agronomy, Lilly Hall of Life Sciences, Purdue University, USA

[3]Institute of Environmental Biotechnology, Graz University of Technology, Graz 8010, Austria

[4]Department of Agroecosystems, Environment and Production, ISARA, Lyon Cedex 07, France

[5]School of Biological Sciences, Faculty of Environmental and Life Sciences, University of Southampton, SO171BJ Southampton, United Kingdom
[6]Present address: International Institute of Tropical Agriculture (IITA), P.O. Box 30772-00100, Nairobi, Kenya * Corresponding Author: Frank Rasche (f.rasche@cgiar.org)

Description
Figure S5: Differentially abundant recruited ASVs between endosphere and rhizosphere compared across Kernza and annual wheat, visualized at a log2 fold-change threshold of 5 (A), and the mean relative abundance of recruited ASVs compared between Kernza and annual wheat (B). Endophyte data used was taken from Michl et al. 2024.

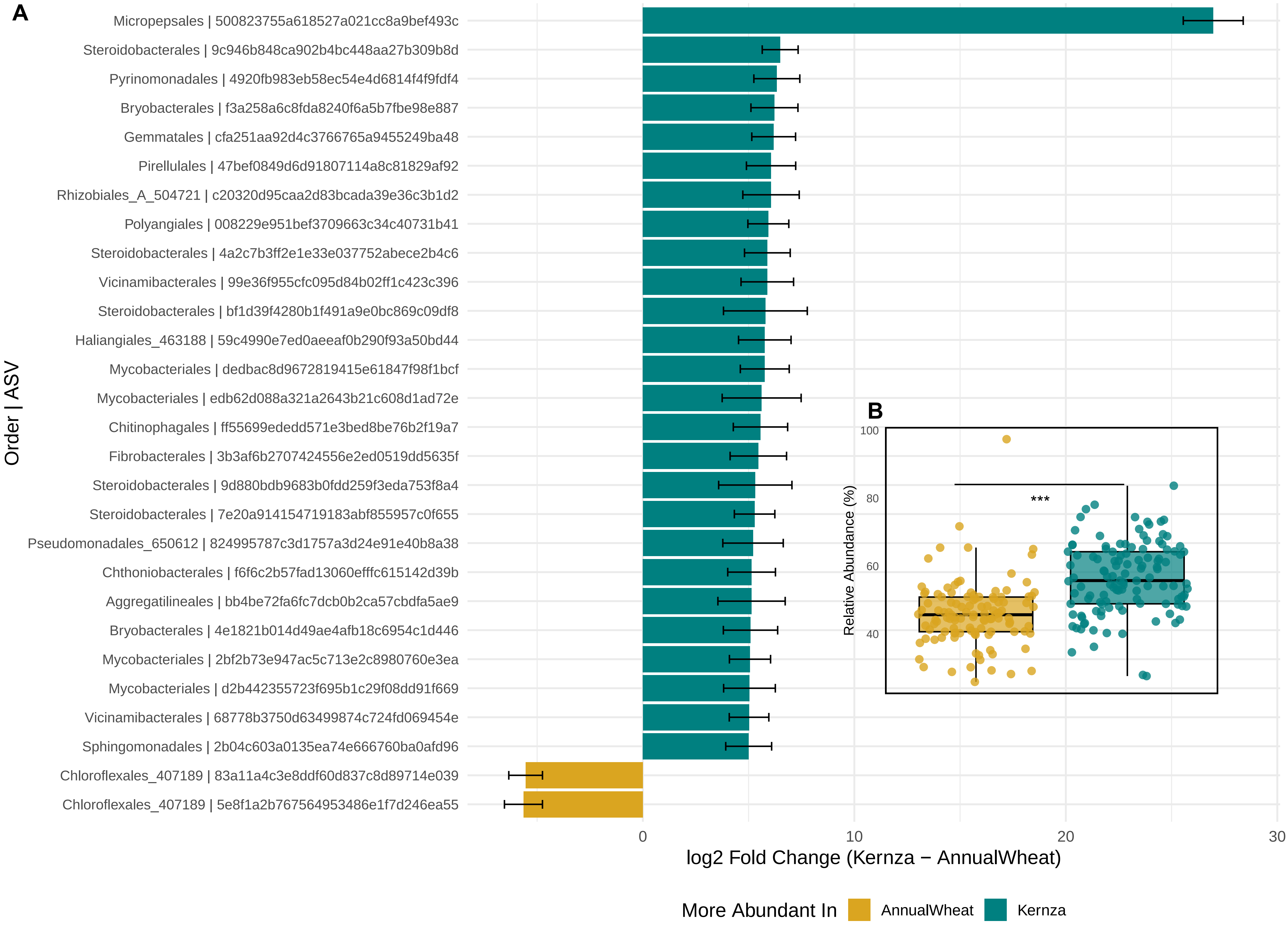
A
Micropepsales | 500823755a618527a021cc8a9bef493c
Steroidobacterales | 9c946b848ca902b4bc448aa27b309b8d
Pyrinomonadales | 4920fb983eb58ec54e4d6814f4f9fdf4
Bryobacterales | f3a258a6c8fda8240f6a5b7fbe98e887
Gemmatales | cfa251aa92d4c3766765a9455249ba48
Pirellulales | 47bef0849d6d91807114a8c81829af92
Rhizobiales_A_504721 | c20320d95caa2d83bcada39e36c3b1d2
Polyangiales | 008229e951bef3709663c34c40731b41
Steroidobacterales | 4a2c7b3ff2e1e33e037752abece2b4c6
Vicinamibacterales | 99e36f955cfc095d84b02ff1c423c396
Steroidobacterales | bf1d39f4280b1f491a9e0bc869c09df8
Haliangiales_463188 | 59c4990e7ed0aeeaf0b290f93a50bd44
Mycobacteriales | dedbac8d9672819415e61847f98f1bcf
Mycobacteriales | edb62d088a321a2643b21c608d1ad72e
Chitinophagales | ff55699ededd571e3bed8be76b2f19a7
Fibrobacterales | 3b3af6b2707424556e2ed0519dd5635f
Steroidobacterales | 9d880bdb9683b0fdd259f3eda753f8a4
Steroidobacterales | 7e20a914154719183abf855957c0f655
Pseudomonadales_650612 | 824995787c3d1757a3d24e91e40b8a38
Chthoniobacterales | f6f6c2b57fad13060efffc615142d39b
Aggregatilineales | bb4be72fa6fc7dcb0b2ca57cbdfa5ae9
Bryobacterales | 4e1821b014d49ae4afb18c6954c1d446
Mycobacteriales | 2bf2b73e947ac5c713e2c8980760e3ea
Mycobacteriales | d2b442355723f695b1c29f08dd91f669
Vicinamibacterales | 68778b3750d63499874c724fd069454e
Sphingomonadales | 2b04c603a0135ea74e666760ba0afd96
Chloroflexales_407189 | 83a11a4c3e8ddf60d837c8d89714e039
Chloroflexales_407189 | 5e8f1a2b767564953486e1f7d246ea55
Order | ASV
0
10
20
30
log2 Fold Change (Kernza − AnnualWheat)
More Abundant In
AnnualWheat
Kernza
B
100
80
60
40
Relative Abundance (%)
***
