# Supplementary material for: Perennial Kernza cropping promotes rhizosphere microbiome stability and endophyte recruitment compared to annual wheat
Source: Environ Microbiome. 2025 Nov 7;20:139. doi: 10.1186/s40793-025-00794-3 (PMC12595868; doi:10.1186/s40793-025-00794-3)
Supplement: Supplementary file 11 — Supplementary Material 11 [file 40793_2025_794_MOESM11_ESM.docx]

**Perennial Kernza Cropping Promotes Rhizosphere Microbiome Stability and Endophyte Recruitment Compared to Annual Wheat**

Sulemana Issifu^1,2^, Arval Viji Elango^2^, Kristina Michl^3^, Christophe David^4^, Tomislav Cernava^3,5^, Roland C. Wilhelm^2^, and Frank Rasche^1,6^

^1^Institute of Agricultural Sciences in the Tropics (Hans-Ruthenberg-Institute), University of Hohenheim, Garbenstr. 13, 70599, Stuttgart, Germany.

^2^Department of Agronomy, Lilly Hall of Life Sciences, Purdue University, USA

^3^Institute of Environmental Biotechnology, Graz University of Technology, Graz 8010, Austria

^5^Department of Agroecosystems, Environment and Production, ISARA, Lyon Cedex 07, France

^6^School of Biological Sciences, Faculty of Environmental and Life Sciences, University of Southampton, SO171BJ Southampton, United Kingdom

^7^Present address: International Institute of Tropical Agriculture (IITA), P.O. Box 30772-00100, Nairobi, Kenya

* Corresponding Author: Frank Rasche ([f.rasche@cgiar.org](mailto:f.rasche@cgiar.org))

**Running Title**: Influence of perennialization on rhizomicrobiomes

**Keywords**: perennialization, rhizosphere, bacterial community, endophytes, annual wheat, Kernza, cropping system microbiology.

**Table S6**. A comparison of rhizomicrobiome diversity and relative abundance between and within crop types at depth

| Comparison | Depth (cm) | Shannon | Padj-value | Rel. abundance (%) | Padj-value | Figure |
| --- | --- | --- | --- | --- | --- | --- |
| Core ASVs (kernza) | 5 - 15 | 3.02 | 0.34 | 0.616 | 0.66 | 5A, 5B |
|  | 25-35 | 3.04 |  | 0.606 |  |  |
| Core ASVs (annual wheat) | 5 - 15 | 2.89 | <0.0001 | 0.690 | 0.66 | 5A, 5B |
|  | 25-35 | 2.36 |  | 0.646 |  |  |
| Kernza vs. annual wheat (Core ASVs) | 5 - 15 | 3.02 / 2.89 | 0.003 | 0.616 / 0.690 | 0.38 | 5A, 5B |
|  | 25-35 | 3.04 / 2.36 | <0.0001 | 0.606 / 0.646 | 0.57 |  |
| Indicator ASVs (kernza) | 5 - 15 | 3.32 | 0.006 | 0.0095 | 0.75 | 5D, 5C |
|  | 25-35 | 2.45 |  | 0.10 |  |  |
| Indicator ASVs (annual wheat) | 5 - 15 | 2.31 | 0.006 | 0.0076 | 0.50 | 5D, 5C |
|  | 25-35 | 3.17 |  | 0.0088 |  |  |
| Kernza vs. annual wheat (Indicator ASVs) | 5 - 15 | 3.32 / 2.31 | 0.0008 | 0.0095 / 0.0076 | 0.87 | 5D, 5C |
|  | 25-35 | 2.45 / 3.17 | 0.019 | 0.10 / 0.0088 | 0.87 |  |
